# Uncertainty, Existential Immobility and Well-Being: Experiences of Women Seeking Asylum in Norway

**DOI:** 10.3390/ijerph192215239

**Published:** 2022-11-18

**Authors:** Zubia Willmann-Robleda

**Affiliations:** Center for Intercultural Communication (SIK), VID Specialized University, 4024 Stavanger, Norway; zubia.robleda@vid.no

**Keywords:** asylum seekers, psychosocial well-being, asylum system, Norway, mental health

## Abstract

In recent years, European countries have made their asylum systems increasingly stricter. Norway has been no exception, taking additional measures to tighten its asylum system to make it less attractive to seek asylum. How does the asylum procedure and living in asylum centres influence psychosocial well-being and, in turn, the prospects of incorporation into a new society? This article identifies the main challenges that a group of women face while seeking asylum and living in asylum centres in Norway, and it explores the influence that these challenges have on their mental health and well-being. To do this, it draws on ethnographic fieldwork conducted during approximately one year (2017–2018) with nine women—of different nationalities, ages and backgrounds—living in asylum centres in Norway. The analysis shows five main elements of the Norwegian asylum and reception system that result in the main challenges that the women deal with during their wait in the asylum centres. These elements are the wait and uncertainty around their asylum application coupled with the inability to influence their circumstances, the limitations to engage in meaningful activities as well as the financial and mobility limitations imposed by the Norwegian authorities. The interconnections of these five elements make the women often feel powerless, unable to influence their circumstances and feel stuck in the present, unable to plan their future, thus, experiencing high levels of uncertainty and existential immobility (Hage 2009). This, in turn, leads to frustration, apathy and even depression in the women, which can have a negative effect on their future incorporation into the Norwegian society.

## 1. Introduction

In 2015 Norway received 31,141 asylum applications as a result of various conflicts around the world, in particular, the Syrian civil war [1]. Previously Norway had been receiving a stable 10,000 asylum applications per year since the 1990s [2]. As a result of the increasing numbers of asylum applications, the Norwegian government, a right-wing coalition led by prime-minister Erna Solberg, started to implement “measures to tighten [the Norwegian] asylum system to make it less attractive to seek asylum” [3]. Similar increasing restrictive measures were also seen implemented in other European countries’ asylum systems. Taking the increasingly hostile regulations and environment for asylum seekers as a starting point, this article explores the experiences of a group of women seeking asylum and living in asylum centres in Norway while waiting for their asylum application to be processed. In particular, it examines the most challenging elements of the Norwegian asylum and reception system for these women and the influence these have on their psychosocial well-being. The process of seeking asylum is often accompanied by extended waits and high levels of uncertainty. Researchers have shown the detrimental effects of long asylum procedures and detention on the mental health of asylum seekers and refugees [4,5,6,7]. The heightened uncertainty of the asylum process in Norway has been seen to have negative effects on young asylum seekers’ mental stability and their sleeping patters [8]. In addition, it has been argued that the longer the stay is, the worse the effects may be on mental health [9]. Research among children living in Danish asylum centres has shown how the long waiting periods together with the uncertainty that it encompasses often results in existential boredom, manifested in fatigue and depression [10]. There is a significant amount of research conducted on asylum seekers’ and refugees’ experiences, in particular, on their psychosocial circumstances in various parts of the world [4,5,6,7]. However, limited research has focused on understanding which particular elements of the asylum procedure and living in asylum centres have an influence on their psychosocial well-being and eventually on their incorporation into the new society. There is a growing body of research that examines the experiences of people seeking asylum and living in asylum centres in the Nordic countries, where the highly structured reception system has been criticized for creating passivity and dependency among asylum seekers and refugees [11,12,13]. Still, much of the research on the issues of psychosocial well-being of asylum seekers has centred around the mental health of unaccompanied minors seeking asylum [14]. Less attention has been placed on adults, and fewer on women, in particular. This is despite 37 percent of first-time asylum applicants in the EU in 2018 being women [15]. In Norway, around 42 percent of asylum seekers in 2019 were female [16]. Nevertheless, few studies have focused on the experiences of women in asylum centres, particularly in Scandinavia [17,18,19]. In addition, the majority of the extant literature that studied asylum seekers’ and refugees’ mental health takes a quantitative as well as a psychology and psychiatry approach, less are qualitative in-depth studies from the social studies perspective to understand the details of what it is that has a positive or negative effect on their well-being. This article draws on ethnographic fieldwork conducted intermittently during approximately one year (2017–2018) with nine women—of different nationalities, ages and backgrounds—living in asylum centres in Norway. The analysis of this data seeks to contribute in-depth qualitative knowledge on the particular elements of the Norwegian asylum system that has an influence on asylum seekers’ psychosocial well-being and potentially on their later incorporation into the new society. For this discussion, the article draws on the notion of psychosocial well-being in refugee contexts developed by Ahearn and defined as “the ability, independence, and freedom to act and the possession of the requisite goods and services to be psychologically content” [20]. The article explores the particular elements of the Norwegian asylum and reception system in the aftermath of the 2015 “refugee crisis” in Europe, while using the concept of existential immobility [21], in other words, the inability to move forward with one’s life due to lack of agency and control, as a lens to understand the experiences of a group of women seeking asylum in Norway.

The analysis shows four main elements of the asylum and reception system that seem to have a negative influence on the women’s psychosocial well-being. These elements are the uncertainty around their asylum application coupled with the inability to influence their circumstances, limitations to engage in meaningful activities as well as the financial and mobility limitations imposed by the Norwegian authorities. The interconnections of these four elements arguably lead to the women feeling powerless, unable to influence their circumstances and feeling stuck in the present, unable to plan their future, thus, experiencing existential immobility [21]. In the following section the theoretical concepts that used as a lens to understand these women’s experiences is discussed.

### 1.1. Psychosocial Well-Being and Existential Immobility

The term “psychosocial well-being” has, in recent years, become more popular among researchers instead of “mental health”; the former is believed to be a term that is less stigmatising and takes the social dimension further into account [20]. The field of research has moved from understanding mental health beyond just the lack of mental disorders and more recently started to acknowledge the role of social and environmental factors on mental health [22,23,24,25].

The term psychosocial well-being is believed to have been popularised by the World Health Organisation in 1996 when mental health well-being was added to the definition of health as “a state of complete physical, mental, and social well-being and not merely the absence of disease or infirmity” [20] (p. 4). However, this definition is still somewhat vague when it comes to the meaning of well-being. Ahearn [20] draws from the work and definitions of Sen [26] and Dasgupta [27] around the concept of well-being and defines psychosocial well-being in refugee contexts as “the ability, independence, and freedom to act and the possession of the requisite goods and services to be psychologically content” [20] (p. 4). Here, we see the importance that having the agency to act and have control over one’s life circumstances has on well-being. Hassan Hage [21] argues that our perception of well-being is tightly linked to an idea of ‘imaginary mobility’. In today’s fast-paced and progress-oriented world, we commonly perceive that we are leading a good life when we are ‘moving’ from one stage in life to the next, from one position to a better one. When this happens, we usually have a feeling that we are ‘going somewhere’, which Hassan Hage [21] calls existential mobility. Contrarily, if we feel stuck, whether it is in a place or in a situation, this is attributed a negative connotation. Thus, if we do not seem to be moving towards that which we expect or aspire to, we will experience existential immobility. According to Bourdieu “uncertainty about the future is simply another form of uncertainty about what [one] is, [one’s] social being, [one’s] ‘identity’… [At stake] is the question of the legitimacy of an existence, an individual’s right to feel justified in existing as he or she exists” [10] (p. 26). In this article, the above discussions around the concept of psychosocial well-being together with Hage’s [21] idea of existential immobility is used as lenses through which to understand the experiences of a group of women living in asylum centres in Norway.

### 1.2. Context and Background

When seeking asylum in Norway, most asylum seekers are accommodated in reception centres (mottak) while their application is processed. Although the waiting time varies significantly from case to case, those who applied for asylum in 2017 waited an average of 321 days until receiving their residence permit [28]. There are two main types of reception centres in Norway. The “centralised” centres are the most common, and they are usually either one large building or a set of small barracks where the asylum seekers are accommodated by gender or family composition. Usually, single women with children and families are in one building and single men are in a separate one, where, depending on the centre, single applicants share facilities, such as kitchen, bathroom, etc. The “decentralised” centres are usually apartments scattered around the urban or sub-urban centre. Families usually have their own private apartment and single applicants share an apartment, sometimes they even share the same room. The “centralised” reception centres tend to be further away from urban and population centres, either on the outskirts of towns or most usually in the countryside. Due to the isolation of the centre, this makes it particularly difficult for its residents to interact with the local community as well as participate in activities to pass their time [29]. Although asylum seekers are allowed to seek a work permit, there are few that do so as they need to have a proof of their identity, through an ID from their country of origin or a passport, something that a large proportion of asylums tend to not have with them due to the circumstances of flight. Those that do receive a work permit find many obstacles to finding a job and getting paid, due to limited language skills, not having their education certificates approved and not being allowed to have a Norwegian bank account [30]. Previously, asylum seekers could voluntarily attend 175 h of Norwegian language class and 50 h of Norwegian culture class, yet, from the 1st of September 2018 this has become mandatory [31]. The kinds of activities that the centre and the municipality offer for the asylum centre’s residents vary from centre to centre. The activities offered are usually language practice, sports, crafts, etc., and they are usually not more than once a week or even more seldom [32].

In addition, due to the closing of borders and several containment policies in various European countries and in Turkey, the amount of asylum seekers that Norway received in 2016 dropped to 3460, a major decrease from the 31,150 applications it received in 2015 [33,34]. As a result, Norway began closing down asylum centres in 2016 and 2017, which led to asylum seekers being relocated to those few asylum centres that remained in operation; some asylum seekers moved centres several times, some of them spending less than a year in each [16]. If the applicant is granted a temporary residence permit (between one and three years depending on the case), the process of resettlement (bosetting) begins. It can take up to several months (in 2017 the average was seven months), for a municipality to accept the refugee and provide them with accommodation and a place in the introduction programme for refugees. After arriving in the assigned municipality, they are provided with monthly financial support, from which they have to pay their accommodation and other living expenses and in return they have to attend the two-year full-time introduction programme (Introduksjonsprogram for flyktninger) where they learn the Norwegian language, about Norwegian society and gain skills to enter the labour market, take up vocational training or continue their education [32].

## 2. Methodology

This article draws on ethnographic fieldwork with nine women during approximately one year (November 2017 to October 2018) while they were living in asylum centres in Norway. This article is part of a doctoral study that explored the experiences and aspirations of women seeking asylum in Norway and the ways in which they navigated the various stages of arrival and settlement in Norway. The data in this article were collected through an initial in-depth semi-structured interview with each participant. The interviews were conducted either in their accommodations or in a room in the asylum centre office, each participant decided where they preferred to be interviewed. The interviews started with broad and open questions to allow the participant to share whatever they wished to. The following questions related to their background, their life before coming to Norway, their experiences in Norway (in the asylum center and with the asylum system) and their view on their future in Norway as well as their dreams and aspirations.

The interviews were complemented with participant observation, including several informal conversations during recurring visits to the women over the course of several months where we would spend hours in their accommodations, drinking coffee/tea, eating and sometimes running errands, such as going to pick up the post at the asylum centre office or going to buy groceries. By spending time with the participants in their ‘homes’ at the reception centres, I was able to get a small glimpse into their day-to-day activities, challenges and emotions. This allowed me to better grasp their narratives in the interviews and to place them in a wider context. I visited each participant usually once a month, with each visit lasting three hours or more, until they received their residence permit and were resettled to a municipality.

Since I wanted the interlocutors to have room to tell their story as they preferred, as well as make them feel free to share anything they wished to, I used a life-history approach [35]. This was carried out with the awareness that stories are not told in a vacuum, as the story teller is influenced by the “biographical and historical moment(s) and (…) (the) prevailing cultural conventions surrounding storytelling, the social context of narration and the audience for a telling” [36] (p. 162). Furthermore, this study draws on the notion of critical phenomenology [37,38], which allows to examine how the socio-political and judicial aspects of being an asylum seeker affects the participants’ everyday lived experiences.

The fieldwork was conducted in two asylum centres in the same region of Norway; these were the only two asylum centres left in the region at the time (2017–2018). At the time of conducting fieldwork there were approximately 30 asylum centres left in Norway [16]. Many centres were closing down due to reduced numbers of arrivals. The women were mainly recruited through the asylum centres, from which I had to receive permission to conduct fieldwork there. The snowball method was tried but it did not seem to work in the context of the asylum centres I was in contact with. Instead, I asked the directors or other employees of the centres to put me in touch with women that fit the criteria. The main criteria was that they either had already received a residence permit or that they had high probabilities of receiving one (given their country of origin or circumstances) so that I could follow the women as they went through the different phases of arrival. After explaining the purpose of my research, I would then ask each woman individually if they were interested in participating. Most women that were approached agreed to participating in the study. There were three that decided not to participate, two I believe was due to the language barrier, which made it difficult for me to explain what the research was about. They may have been scared or unsure about who I was and what the purpose of me talking to them was. The third one had to travel often to take care of her family and for practical reasons she did not end up participating in the study. From the women that participated in the study, I experienced that most of them saw the value of sharing their experiences with me as a way to let their struggles and challenges be heard or read by others to hopefully contribute to improving the situation for other asylum seekers in the future.

Given that I primarily used the help of the asylum centre employees to recruit the participants of this study, the sample selection could, thus, have been influenced by the asylum centre employees. They may have acted as gatekeepers, suggesting for me to talk to women that they may have a better relationship with and, thus, make it more difficult for me to reach other women that maybe had little or a bad relationship with the centre’s employees. This could have resulted in having different kinds of data and results.

After six months of challenging recruitment, I ended up managing to recruit nine women, aged between 23 and 45 years from various national, ethnic and religious backgrounds as well as different educational levels and family circumstances. The majority were from countries in the Middle East or East Africa, seven of the nine were Muslim, although from different branches and traditions, and had arrived in Norway in 2016–2017. Some had children and their husbands with them, others came alone. When I first met them, some participants had already received a residence permit while others were waiting to have their asylum application processed. The sample size is small and, thus, not aimed at achieving any representativeness to generalise results for all women seeking asylum in Norway. Nevertheless, what the study aims to achieve is an in-depth understanding of the asylum-seeking experience in Norway and its relationship with mental health. Previously, research, especially research on mental health issues among asylum seekers, has been quantitative and tended to show the general trends but not so much their in-depth narratives and experiences. The interviews and notes from observations and informal conversations were transcribed verbatim and consequently thematically analysed and coded according to major themes emerging across the data. The use of the software NVivo was used to organise and code the data material in a systematic and organised way.

Given the sensitive nature of the research and the ‘vulnerable’ circumstances that the participants were in, several ethical concerns were taken into consideration. At the time of the initial interview, many participants had still not received an answer to their asylum application; hence, I did my best to explain the purpose of the research and that participating in the study would not affect their asylum application. I made sure they understood my role as a researcher and interviewer (who was also a female) and that they would remain anonymous to anyone else but me and the occasional interpreter, who also signed a confidentiality form. After making sure they understood this and gave their consent to participate, they were asked to sign an informed consent form, while being informed that they could refuse their participation at any time.

Finally, I managed to build a certain rapport with most of the women because I am not Norwegian and I am also an immigrant in Norway; I also had to learn the language and navigate the new societal codes as well. This may have made the women more comfortable around me and share maybe more critical stances towards their experiences in Norway than if I had been Norwegian. This, however, comes with an important caveat; I am a European citizen, and I could be confused for being Norwegian due to my appearance; hence, my experiences as an immigrant in Norway are rather different from those of these women, yet there are some elements that I believe brought some of us closer. There are of course other factors, such as language, age, level of education and life experience that made certain women feel more comfortable and create further rapport with me as compared to others.

### 2.1. Uncertainty, Existential Immobility and Well-Being in the Asylum Centre

#### 2.1.1. Long Waits and Uncertainty

It is a cold and rainy winter day, and I am having a strong Arabic coffee while sitting in an old leather sofa in a dimly lit room that serves as a living room during the day and a bedroom at night. I am visiting Maryam, who came to Norway in late 2016 from a country in the Horn of Africa. She has been living in this asylum centre for the last nine months after being relocated from a transit centre after her asylum interview. She shares the studio apartment with Nayyat, a young woman from Ethiopia, who has now become her close friend. The studio of approximately 15–20 square metres consists of bathroom, kitchenette, a room with a bunk bed and a living room. Nayyat sleeps in the room with the bunkbeds, while Maryam sleeps on the sofa in the living room, that way they have at least a bit of privacy at night, as they say. The studio is in what looks like an older prefabricated building, part of an asylum centre in the industrial area outside of a small town, and this is where they spend most of their days. Waiting. This is the third or fourth time I am visiting them, and we are having a conversation after having delicious food they made, which is how they always greet me. Nayyat retires to pray while Maryam and I start talking about the challenges they face in Norway while seeking asylum.

“The worst thing in Norway is they do not tell you that you will get it (the answer to the asylum application) in three months, in one year or in one and half years. They just say: ‘Wait’. It is not something you can live with easily”.

(Maryam)

This is not the first time that Maryam mentions the arduous waiting and she is not the only one either. This is also one of the most common topics of discussion with other women I talked to. For Maryam, as for many other women, it is not just the waiting per se, but the seemingly endless wait coupled with not knowing when it would end or what would be the outcome of such a wait that made it so challenging. They were all hoping to get their asylum application approved and be able to stay in Norway and start their lives there, but this was not a given for all. There was a chance for all that their asylum application would not be accepted and, thus, they would have to either appeal and wait again for the answer to this, or leave the country, in the worst case even have to return or be deported to the place they fled from in the first place.

Coupled with this is what many also mentioned as their inability to do much about it. They described feeling powerless and outraged that there was nothing they could do to influence their situation, as they did not feel like their destiny was in their hands. Pınar, a mother of two from Turkey, tried to explain how she felt the authorities’ narrative was about the wait. ‘“You are here in this room and I will give you as much money as I want, you shouldn’t call me all the time, don’t disturb me, just wait”’. She and her husband used to call The Norwegian Directorate of Migration often, but their answer was always the same: “Don’t call again…just wait”. Pınar felt it was unfair how the authorities would not provide her and her family with information about their case and they were simply to wait and not to get in contact. As Pınar explains, asylum seekers are at the mercy of the authorities, unable to take control of their situation. Many women mentioned that this uncertainty, limited ability to do much about their current situation, long periods of open-ended waiting and seeing other refugees get answers faster than them, affected their mental health. As Nayyat explains:

“I have been here now for three years. I waited one year until I had the interview, after that they told me I would only wait a few months because I already waited a year. I waited six months. I waited more. One year and eight months afterwards they gave me an answer. Other refugees that had come after me, they left the asylum centre before me. This is bad for your mental health, it adds to your previous problem, this is very hard”.

(Nayyat)

Here, we see that Nayyat also points to something that other women also mentioned, that is that the uncertainty and other challenges of the asylum-seeking process, as we will see, added to previous traumas or problems they had from before, and led them to spend more time ruminating about these. Previous research on asylum seekers has also highlighted the long waiting times and high levels of uncertainty that the asylum process is characterised by [8,39]. These studies have even raised the issue of how long waiting times in asylum centres can potentially aggravate psychological issues that asylum seekers may already have from before. Indeed, Sveaas [40] believes that more than half of those that get residence permits in Norway have psychological issues that may require treatment. However, scholars have also highlighted that not enough attention is placed on mental health during the asylum process and even later on during resettlement [41,42].

#### 2.1.2. Limitations to Engage in Meaningful Activities

Many women mentioned that the seemingly endless wait and the feelings of powerlessness were aggravated by the limited number of meaningful activities they could engage in while waiting. As mentioned earlier, asylum seekers face significant barriers to acquire a work permit and consequently employment. One of the few activities they can engage in is to attend the 175 h of Norwegian lessons they have a right to, and the occasional activities provided by the asylum centre or local civil society organisations, such as knitting, language practice, sports and a few others. A significant barrier to being able to engage in meaningful activities while living in asylum centres is the location of the centres, usually placed far away from population centres, in rural or industrial areas. A growing body of research on asylum centres has brought the issue of their physical isolation, and as a result social isolation, to light and the way this negatively affects the well-being of their residents [12,19,29].

In this study, most women were highly concerned about the lack of meaningful activities for them to do in their daily life in the reception centre while waiting. Nousaiba, a young woman from Sudan, explained it as follows:

“Here, there is nothing else than to eat and sleep. (…) I became so exhausted, so tired, I have been so much time in this room [in the asylum centre]. I cried; I am alone. During a long time, I did not go to class, I had no one to talk to, I was depressed. I am also a human that has feelings … and I think too much. I began to think about my family, how they died, what happened. I did not have anything to do, so I began to remember things”.

(Nousaiba)

The limited possibilities to engage in meaningful activities, resulted in them spending long hours just sitting in their small rooms, which contributed to thinking more about their situation, which, as Nousaiba mentioned, often led to replaying traumatic memories and, thus, worsened mental health and significantly affected well-being. This led them, in turn, to feel apathetic and passive and not have energy or motivation to go to class or engage in any other activity, which contributed to making the wait even more endless. For many like Nousaiba, the activities that they could engage in often seemed meaningless, they were just a way to fill the time, to distract them from waiting, which at times was useful, yet at other times felt pointless. Recent research conducted in The Netherlands on asylum seekers, their length of stay in asylum centres and the link to mental health, shows that despite the centres implementing increasing day time activities, this seemed to have limited effect on the asylum seekers’ mental health and well-being [43]. This correlates with this study’s findings and what Nousaiba points at above, about how often the activities they undertook to fill the time felt pointless and contributed little to their general well-being; particularly, for the women who stayed longer at the asylum centre, the less interest they had in spending their time participating in any activities.

Norwegian class was often mentioned as the most meaningful activity as it was one that made them feel like they were gaining some skills and moving forward, (hopefully) preparing for their life to start.

“I don’t do anything, if I don’t have school (Norwegian lessons), there is nothing to do. I had school for example last week Tuesday. On Wednesday I have swimming. On Thursday I have also school but nothing else. Sometimes if I am busy, I have these things it will be a busy week, three things in three days, so many! (uses an ironic tone). But now I don’t have anything this week only Thursday, Tuesday I don’t have school, so nothing else”.

(Maryam)

For many like Maryam, Norwegian class seemed to provide them with a routine and gave them a purpose while waiting for their answer. Pınar expressed that attending these classes would bring her “back to life”. Previous research has also identified Norwegian class as one of the few activities people in asylum centres are able to participate in, thus, providing them with a routine and a purpose [44]. However, this research has not discussed the barriers that asylum seekers often encounter in attending the Norwegian lessons. Most asylum centres in Norway, and like the ones where the interlocutors lived, are rather isolated from urban centres, as mentioned above. The women I talked to mentioned facing obstacles in attending Norwegian class as it was in the town centre and they had to take a bus, which was not covered by the government support. In one of the centres where some of the women lived, the Norwegian classes were more than a thirty-minute walk away in the usually cold and rainy or snowy weather. As Maryam expressed: “With the cold it’s really hard, and it’s far away and it’s not economic to go by bus, every time it’s 37 NOK (approximately EUR 4) for each way so it’s really hard”. Hence, we can see that the location of asylum centres, often isolated from population centres, becomes an obstacle for asylum seekers to partake in Norwegian classes, one of the few meaningful activities that can provide them with some routine and purposeful distraction. We can also see how this activity may also provide them with a sense of moving forward in their lives, which is existential mobility [21] and, thus, positively influence their overall well-being while seeking asylum. That is because many women generally saw learning Norwegian as a useful activity to spend their time on as it was a skill that they would need if they were granted asylum and eventually stayed in Norway. Other activities (such as knitting or sports) may have felt less useful and meaningful and just a trivial thing to do to pass the time.

As mentioned above, asylum seekers also face several challenges in attaining a work permit and, consequently, to finding a job while they wait for the answer to their asylum application. The work permit can be attained once proof of identity is provided, something that is difficult for many asylum seekers fleeing without any documents. Yara, a single young woman from Syria, was the only one among the women I talked to who had applied for and received a work permit. However, she had problems finding a job, even finding a volunteering job proved to be difficult. This can be, in part, the result of her limited Norwegian skills but also because the asylum centre is in a rather isolated location, where job opportunities are not abundant. Many, like Yara, mentioned how challenging it was for them not to be working or doing much for such a long time.

This limited possibility to engage in meaningful activities exacerbated the women’s feelings of ‘stuckness’ and lack of control over their present situation and destiny [21], which is visible in Yara’s following quote: “After getting the residence (permit) our life will start”. She explained how life in the asylum centre was a “life on hold”. This was because they had to wait for a decision to be taken on their asylum case, and because of the limited possibilities to engage in meaningful activities that allowed them to feel like they were moving forward in their life, as we have seen above.

Furthermore, I observed that the extended uncertainty and this feeling of their lives being on hold seemed to lead most of the women to feeling depressed, demotivated and fall into a state of passivity. Pınar explained it in her own words, “As time goes by, you have less hope, and your will to do something also decreases”. The more she waited, the less hope she had and, thus, the less motivation she had to do anything. She explained how she lacked the energy to do more than just what she needed to, which was to take care of her children, the rest of the day while they were in school, she would often just sit in the room without doing much. She further explained:

“I have been here for almost two years. I stopped dreaming for six months (…) six months ago or a year ago, if the country had given me a residence permit, I could have been a stronger person psychologically. I would have tried harder to learn the language”.

(Pınar)

For Pınar, having such an uncertain future resulted in lacking motivation to learn Norwegian. Other researchers have also found that uncertainty over their lives can impact refugees’ motivation to integrate and learn the host country’s language in that they may be unsure of the purpose of learning the host country’s language [8,45,46].

Nayyat also described not seeing the point in participating in any activities after some time.

“I love studying and knitting but with time you say what is the point? You know? There is nothing. You don’t have an answer, you think all kind of thoughts, you don’t know what will happen to your future so what is the point of doing these things”.

(Nayyat)

She had lost the purpose and meaning, leading her to have no motivation to attend the few activities available due to the lack of certainty about her stay in the country, thus, adding to the feeling of “stuckness” and existential immobility. This echoes previous research that has shown that longer stays in asylum centres significantly weakened asylum seekers’ mental health [43]. A similar pattern has been found among individuals in immigration detention in the UK [47]. This shows that the experience of seeking asylum and living in an asylum centre is not that different from being in immigration detention. Thus, it may be argued that making the asylum-seeking process this challenging may be part of the state’s goal of making the asylum system less attractive to potential asylum seekers, as mentioned at the beginning of this paper. We can see some similarities between the findings of previous research in immigration detention centres where “immigration detainees live in a context of continual crisis, in which profound uncertainty becomes normalised. This disorder should be understood as a technique of power, with governance through uncertainty constructing certain immigrants as expendable, transient and ultimately, deportable” [48] (p. 263).

#### 2.1.3. Financial Limitations and Forced Spatial (Im)mobility

In many of the women’s narratives, there were two further elements of the reception system that seemed to exacerbate their feelings of being “stuck” or unable to move forward with their lives, thus, significantly influencing their well-being. This was the limitations to their mobility both within Norway and internationally, as well as the limited financial support provided by the government coupled with their limited financial means.

Several women mentioned that the financial support they received by the Norwegian state while living in asylum centres was barely enough to cover basic food necessities. Sometimes they needed to buy clothes, especially for the cold winter months, as many had come with what they had on or a small bag, yet the money they received was often not enough to do so. This bimonthly allowance was often not enough for them to pay for public transportation on a regular basis. This, as we have seen above, had an impact on their ability to even attend Norwegian classes in the town centre, which as we have seen was one of the few meaningful activities they had the possibility to undertake. In addition, many asylum seekers have little money of their own, either because they have left all their assets behind and have no way to receive money in Norway as they are not yet able to open a bank account there or because they have spent it to pay for their journey to Norway. This makes them dependent on the financial assistance of the state [11,18]. Thus, several women like Yara mentioned that their life felt like it was on hold also due to their dire financial situation and the limited financial support they received from the state, which meant that they could do little more than “eat and sleep” while they waited, as some women mentioned. This echoes previous research among asylum seekers in the Nordics, such as Iceland, where asylum centre residents also explained that they had “nothing to do”, for them, similar to the women I talked to, having something to do meant having employment, and they also found that a lack of employment significantly affected their self-image and well-being [48].

In addition to these financial limitations and, thus, dependency on the Norwegian state, the women often mentioned the limitations that the Norwegian state put on their mobility, both nationally and internationally, as another element that made them feel stuck. While living in asylum centres, asylum seekers are expected to live in the asylum centre full-time to be provided with the financial support that it comes with. Travelling internationally is completely out of the question as their personal documents (if they have any) are kept by the Norwegian Directorate of Immigration while they process their asylum application. If they wish to travel and spend the night elsewhere in Norway, for example, to visit family and friends, even if it is in the same city as the asylum centre, they must fill out a form stating their reasons for requesting this, which is then processed by UDI. There is also a limit as to how many days they can be away from the asylum centre at a time. For those that had friends and/or family in other parts of Norway, such as several of the women, this was challenging. Yara was particularly bothered by this, she had a sibling who had come to Norway before her and lived in a different municipality several hours away and she liked to visit him often as a way to get out of her small room in the asylum centre.

“When I want to go to see my sibling (living in another part of Norway) I have to ask the office and they send the paper to UDI and they will think about why I have to go to see my sibling, if it’s not a good reason they say no, so you… (laughs nervously) you feel like a baby, like I have to take the answer from my father and mother…it’s difficult”.

(Yara)

She felt like with these measures, asylum seekers were treated like small children who were not free to make their own decisions but had to ask permission from their parents (in this case the state) to do almost anything. We can see how this, which can be considered to a certain extent forced spatial immobility imposed by the Norwegian state as part of the asylum reception system, reduces the agency that asylum seekers have. It made women feel frustrated and exacerbated their feelings of being physically stuck and unable to move forward with life. Thus, we can observe how having limited agency, which is limited control over one’s life, has a negative impact on a person’s well-being. Although asylum seekers are not living in a closed centre and to a certain extent have the possibility to leave the centre, as we have seen, they still experience a significant number of limitations to their mobility, and the effects of these are similar to those experiences among immigration detainees [47].

In the women’s narratives, I could also observe their agency being constricted through forced spatial mobility. As we have seen in the context section, asylum seekers are first placed in an arrival or transit centre and then moved to a regular asylum centre. Especially since the end of the European “refugee crisis”, many were made to move from one asylum centre to another, given the closing down of several centres in Norway. Thus, we can say that some women not only experienced limited possibilities to move freely wherever they wanted, but they also experienced being forced to move from one asylum centre to another due to the closing down of centres mentioned earlier in the context section. Several of the women in this study experienced being moved from one centre to another several times while waiting for either their asylum application or their resettlement to a municipality to be processed, as Pınar explains:

“While I was in class in the other asylum centre, we got the news that the camp was going to close. That is the worst thing you can do to asylum seekers. It is already hard enough to get used to one place. And they will tell you: ‘You will go away from here too’”.

(Pınar)

Similarly, Haya, a Syrian mother of four mentioned: “I have been suffering so much and it’s been very hard for me, especially when everything that I have to do is prepare the clothes and the luggage and move to another camp”. As Pınar and Haya indicate, it was not only the fear of getting a rejection to their asylum claim and then being potentially deported that affected their mental health, but also the constant moving, which aggravated the lack of stability they had in their lives as they did not know how long they would stay in each reception centre. Furthermore, as the women’s quotes depict, this forced spatial mobility made their everyday lives more unstable by making them feel further uprooted. This forced spatial mobility could be argued to further add to their feelings of being unable to take control of their lives as it further limited their ability to plan their future, thus, adding to their feelings of existential immobility [21] and negatively affecting their well-being. Previous research has also shown that forced relocations weakened refugees’ mental health [49], as it led to feelings of unpredictability and powerlessness [43,50].

Furthermore, the constant moving made it difficult for the women to keep a routine and to build up a social network that would support and help them when needed. Social networks are said to play an important role in the resilience and well-being of refugees and asylum seekers [32,45,51]. As Pınar put it, to move each time would mean to lose the social network they had built and to have to get used to a new place, not knowing what they could expect in the next location. In particular, the women that had their children with them found it especially challenging to move centres again and again as this meant that their children had to leave their friends behind and start school or kindergarten somewhere else.

#### 2.1.4. Wasted Time

Finally, several women expressed that they felt that the time they had spent waiting in the asylum centres was wasted time, something that they could not recover and that could significantly affect their lives after leaving the asylum centres, which at times seemed to exacerbate their depression. We see it here in the words of Nayyat:

“Maybe it [the wasted time in the asylum centre] will affect [me in the future], because already you are older now, three years have passed, you could have been studying and finish your studies so many things I could have done, they are gone already, so maybe, you don’t know, this is the hard things in the mottak (asylum centre), you never forget it. I can’t forget it, ever”.

(Nayyat)

Pınar was even more pessimistic about it: “…even if I get the residence permit, we missed a long time, we cannot adapt back to life…”. Thus, according to these women, the experience in the asylum centres waiting and wasting time may even have a damaging effect on their later adaptation to life in Norway after resettlement. The women’s worries are in line with findings in previous research that show that long waits on asylum claims with high uncertainty has a negative effect on asylum seekers’ and refugees’ subsequent labour market participation [52]. This can be both attributed to the pause in their career but also to the impact that long asylum procedures tainted by high levels of uncertainty have on mental health, which may still affect asylum seekers after being granted asylum and getting ready to look for work [53,54]. Further research on this issue is warranted.

## 3. Conclusions

This article has explored the everyday lived experiences of nine women seeking asylum and living in asylum centres in Norway and discusses the effects of the five main elements of the Norwegian asylum and reception system on their psychosocial well-being. These five elements are the seemingly endless wait and uncertainty, limited ability to control their circumstances, the limitations to engage in meaningful activities, financial limitations and forced spatial (im)mobility.

The women pointed to the arduous and uncertain wait for their asylum claim to be processed as the main challenge of seeking asylum in Norway. This, together with the limited information they received from the Norwegian authorities about the process of their asylum case, as well as the uncertainty of what the answer to their asylum claim may be, was described as extremely difficult. In addition, the women had limited possibilities to change their circumstances, all they could do was wait, without knowing how long or for what exactly they waited. This challenge of the uncertain waiting and feelings of powerlessness was coupled with the limited possibilities of undertaking meaningful activities while they waited, adding to time spent dwelling over previous traumas. They faced barriers to employment, they had a limited number of hours of Norwegian lessons and there were few other activities to fill their time. This exacerbated the women’s feelings of lack of control over their present and future leading them to feeling stuck.

The limited financial support that the state provided them while waiting on their asylum claim further narrowed possibilities of engaging in meaningful activities, as this was often not enough to even take public transportation. Thus, this affected their chances of attending Norwegian classes and other courses to make them more employable or to use the waiting time in a meaningful way. A further element that added to their feelings of being stuck, in this case physically, was the limited freedom they had to travel within Norway, and this was not at all possible internationally. Simultaneously, several of the women were also forced to move asylum centres several times while waiting for their asylum claim, thus, adding to their inability to control their circumstances and making it difficult to plan ahead. This juxtaposition of simultaneous forced spatial immobility coupled with forced spatial mobility, added to their feelings of powerlessness during their uncertain waiting process.

All this made the women feel unable to move forward with their lives as they were provided with limited opportunities to do so; hence, their possibilities to take control of their current and future situation also felt limited. Thus, through the women’s narratives, the article has shown how the elements of the Norwegian asylum and reception system seem to have a significant influence on asylum seekers’ psychosocial well-being. It has further discussed how the uncertain wait together with the other four elements of the Norwegian asylum and reception system seems to induce powerlessness and feelings that their lives have been put on hold, thus, leading them to experiencing existential immobility. The women’s narratives above also show that such feelings of existential immobility and powerlessness lead many to become depressed, demotivated and worried about the effect the ‘wasted time’ (as many described the time in the asylum centres) may have when or if they get to start their new lives in Norway if their asylum claim is accepted. This coincides with previous research on Nordic asylum and reception systems that has criticised these for being highly structured, leading to passivity and dependency among asylum seekers and refugees [11,12,13]. This article has sought to contribute to this body of literature by zooming in on the experiences of women seeking asylum, a group that has gotten significantly less attention in the previous literature, and provide a better understanding of what elements of the Norwegian asylum and reception system influences asylum seekers’ psychosocial well-being.

These findings are qualitative and, thus, cannot be generalized; however, they do provide us an in-depth insight into asylum seekers’ experiences, which can aid both health and social service providers whenever working with such a group. Understanding the elements that have an impact on the psychosocial well-being of asylum seekers and refugees can be useful for these health and social service providers, particularly when they are enroled in integration programmes. Experiencing challenges to their psychosocial well-being during the asylum seeking procedure (as well as before arrival) can have a significant impact on asylum seekers’ and refugees’ incorporation process into a new society [52], therefore, grants further exploration on a larger scale to better understand such a relationship.
